# Contactless multiscale measurement of cardiac motion using biomedical radar sensor

**DOI:** 10.3389/fcvm.2022.1057195

**Published:** 2022-12-13

**Authors:** Jia-hao Qiao, Fu-gui Qi, Fu-lai Liang, Jin Ma, Hao Lv, Xiao Yu, Hui-jun Xue, Qiang An, Ke-ding Yan, Ding Shi, Yong-hui Qiao, Jian-qi Wang, Yang Zhang

**Affiliations:** ^1^Department of Military Biomedical Engineering, Fourth Military Medical University, Xi'an, China; ^2^School of Electronic Information Engineering, Xi'an Technological University, Xi'an, China; ^3^School of Aerospace Medicine, Fourth Military Medical University, Xi'an, China; ^4^State Key Laboratory of Clean Energy Utilization, Zhejiang University, Hangzhou, China

**Keywords:** radar cardiogram (RCG), cardiac motion, anteroposterior measurements, along-the-arc measurements, diastasis measurement

## Abstract

**Introduction:**

A contactless multiscale cardiac motion measurement method is proposed using impulse radio ultra-wideband (IR-UWB) radar at a center frequency of 7.29 GHz.

**Motivation:**

Electrocardiograph (ECG), heart sound, and ultrasound are traditional state-of-the-art heartbeat signal measurement methods. These methods suffer from defects in contact and the existence of a blind information segment during the cardiogram measurement.

**Methods:**

Experiments and analyses were conducted using coarse-to-fine scale. Anteroposterior and along-the-arc measurements were taken from five healthy male subjects (aged 25–43) when lying down or prone. In every measurement, 10 seconds of breath-holding data were recorded with a radar 55 cm away from the body surface, while the ECG was monitored simultaneously as a reference.

**Results:**

Cardiac motion detection from the front was superior to that from the back in amplitude. In terms of radar detection angles, the best cardiac motion information was observed at a detection angle of 120°. Finally, in terms of cardiac motion cycles, all the ECG information, as well as short segments of cardiac motion details named blind ECGs segments, were detected.

**Significance:**

A contactless and multiscale cardiac motion detection method is proposed with no blind detection of segments during the entire cardiac cycle. This paves the way for a potentially significant method of fast and accurate cardiac disease assessment and diagnosis that exhibits promising application prospects in contactless online cardiac monitoring and in-home healthcare.

## 1. Introduction

In recent years, physiological signal measurements and perceptions have always been among the most popular research topics in auxiliary diagnosis medical technology (1). As the most important organ and the “engine” of the human body, heart status detection undoubtedly holds great significance in human health monitoring. With the development of sensor technology, numerous sensor-based applied studies have been oriented toward cardiac signal measurements. At present, classical cardiac information detection technologies mainly include electrocardiography (ECG) (2) and photoplethysmography (PPG) (3), which are relatively mature and widely used in clinical practice. ECG detects electrophysiological signals generally using metal electrodes placed on the body surface, and PPG detects optical signals at the wrist or finger (which are affected by blood volume changes) through optical sensors. However, both of the above methods require bodily contact, especially ECG, which requires electrode attachment and is not suitable for future round-the-clock real-time home health monitoring and diagnosis monitoring for burn or infected patients. Therefore, if contactless and unconstrained cardiac motion information detection technology can be developed, this would be highly promising to serve as core contactless physiological measurement technology for next-generation smart medical detection and smart home health monitoring.

Bio-radar research originated in the 1970s (4) and has been widely studied over the last 20 years because of its unique advantages. The basic principle of bio-radar for detecting vital signs is that cardiopulmonary activity (heartbeat and respiration) causes micro-movements of the body surface. These micro-movements generate specific modulation of the electromagnetic waves transmitted by radar sensors which are then reflected. Consequently, human heartbeat and respiration signals can be obtained through demodulation operations on radar echoes. More importantly, considering its unique advantages, such as privacy preservation, penetrating nonmetallic obstacle detection, and sensitivity to finer motion, bio-radar technology has been widely applied in vital sign detection (5, 6) and target localization (7), especially in the field of non-contact detection of respiratory and heartbeat signals for broad applications (8–13).

In terms of cardiac motion information detection, in recent years, although many bio-radar-based studies on physiological information (such as heartbeat and respiration) have been carried out, most of these are mainly concentrated on coarse-grained information such as heart rate (14), heart rate variability (2) and other statistical indexes. This coarse-grained information can partly reflect the target's physiological state and health status, but we prefer to obtain more specific and detailed information of cardiac movement similar to the ECG waveform signal, which is more conducive to facilitating deep and careful observation of the time-varying state of heartbeats and even for assessing relevant cardiovascular function and diseases. Unfortunately, only a few exploratory studies have been conducted to date. For example, Aardal et al. (15) stated that the bio-radar was first exploited to detect the two main and detailed cardiac activities of ventricular ejection and filling. Wang et al. (16) used a bio-radar to extract two geometric feature points corresponding to the atrial and ventricular contractions of an atrial-ventricular co-motion simulator. Furthermore, Gao et al. (17) found that time delays between contractions and relaxations of the atrium and ventricle can be observed in radar echoes. In contrast, Zhu et al. and Dong et al. (18, 19) verified that five feature points of radar heartbeat signals detected from the back of the body could be extracted, which consistently corresponds to five points in the ECG. Moreover, four different body orientations for heartbeat signal detection during normal breathing were investigated, and the results showed that the amplitude ratio of the heartbeat to the respiratory harmonic in the frequency domain from the back was greater than that from the front (20).

Generally, preliminary studies have obtained time-varying signals of cardiac motion and principally found corresponding relations between the radar cardiogram (RCG) and ECG in physiological feature points. However, three significant points remain unexplored: (1) The influence of some key factors on the RCG detection, such as the heart anatomical position, posture, and atrial and ventricular motion characteristics; (2) The corresponding relationships between the features of the electrical signal, radar echo signal, and physiological process-oriented cardiac motion; (3) In the ECG signal, there is a period with no electrical stimulation or conduction in a cardiac motion cycle (from the last T-wave to the next P-wave), resulting in a flat waveform, which is called diastasis (21). During diastasis, the ECG does not contain information about cardiac motion, but the heart still undergoes corresponding blood flow movement and volume changes during this period. Therefore, it is worth exploring whether motion-sensitive RCG can detect cardiac motion during this special period.

Based on the physiological characteristic analysis of cardiac three-dimensional motions and the proposal of classical signal processing schemes for RCG, this study designed and implemented multiscale cardiac motion measurement experiments based on the IR-UWB radar system to investigate the three significant points mentioned above. Leveraging the anteroposterior measurements, along-the-arc measurements, and comparative experiments with ECG focusing on diastasis detection, the rationality and advantages of bio-radar for cardiac detail monitoring and cardiovascular disease diagnosis are thoroughly discussed.

This paper is organized as follows: Section 2 introduces the principle of radar cardiac motion detection. Subsequently, the physiological process of cardiac movement alone with three dimensions and the advantages of RCG for cardiac motion detection were analyzed, and the radar sensor and experimental scheme are described. In Section 3, signal processing and feature extraction methods are presented. The experiments and results are presented in Section 4. In Section 5, the discussion and conclusions are presented.

## 2. Materials and protocol

This section contains four parts, including the principle of bio-radar-based heartbeat detection, the three-dimensional motion mechanism of the heart, the advantages of RCG over ECG in heart motion detection, and a corresponding experimental scheme illustration.

### 2.1 Principle of cardiac motion detection based on IR-UWB

The IR-UWB radar acquires physiological information by analyzing the time and amplitude variations of reflected pulses. When the transmitting antenna transmits very short pulses at the carrier frequency to illuminate the human chest, the receiving antenna receives the corresponding reflected electromagnetic wave modulated by the thoracic motion caused by respiratory and heartbeat organ behavior. Consequently, the micro-motion Doppler signal can be derived from (1).


(1) $$\begin{array}{l} s\left(t\right)=d_{0}+d\left(t\right)=d_{0}+d_{r}\sin\left(2\pi f_{r}t\right)+d_{h}\sin\left(2\pi f_{h}t\right) \end{array}$$

where *d*_0_ is the fixed distance between the antenna and human chest wall, *d*_*r*_is the displacement amplitude of respiration, *d*_*h*_ is the displacement amplitude of the heartbeat, and *f*_*r*_ and *f*_*h*_ represent the respiratory and heartbeat frequencies, respectively.

Denoting the normalized received pulse as δ(*t*), the total response can be expressed as follows:


(2) $$\begin{array}{l} r\left(t,\tau \right)=A_{k}\delta \left(\tau -\tau_{k}\left(t\right)\right)+\sum_{i=1}A_{i}\delta \left(\tau -\tau_{i}\right) \end{array}$$

where *t* is the observation time, and τ is the propagation time. where δ(*t*, τ) is the generated short pulse centered at the carrier frequency*V*_*c*_. *A*_*k*_ and *A*_*i*_denote the amplitudes of the target response and the multipath components, respectively, while τ_*k*_(*t*) and τ_*i*_ are the corresponding delays.τ_*k*_(*t*) is determined by the antenna distance to the target, which is expressed as


(3) $$\begin{array}{l} \tau_{k}\left(t\right)=\frac{2s\left(t\right)}{c}=\tau_{0}+\tau_{r}\sin\left(2\pi f_{r}t\right)+\tau_{h}\sin\left(2\pi f_{h}t\right) \end{array}$$

where the speed of light *c* is ~3 × 10^8^*m*/*s*, τ_0_ = 2*s*/*c*,τ_*r*_ = 2*d*_*r*_/*c*, τ_*h*_ = 2*d*_*h*_/*c*_._

Radar converts the received signal into a matrix of *m*rows and *n*columns, denoted as *R*[*m, n*],


(4) $$\begin{array}{l} R\left[m,n\right]=r\left(t=mT_{s},\tau =nT_{f}\right) \end{array}$$

where *m* and *n* represent the sampling numbers of slow time and fast time, respectively. *T*_*s*_ is the pulse duration of slow time, and *T*_*f*_is the sampling interval of fast time. The row vector records the echo signals received at different observation times in each range interval, whereas the column vector records the echo signals received at different distance intervals in each time interval. Conventionally, vital sign information can be extracted by directly applying a Fourier transform to the cross-range slow time samples fixed at the range bin that contains most of the energy from chest movement.

### 2.2 Analysis of physiological characteristics of three-dimensional cardiac exercise

**Figure 1:**
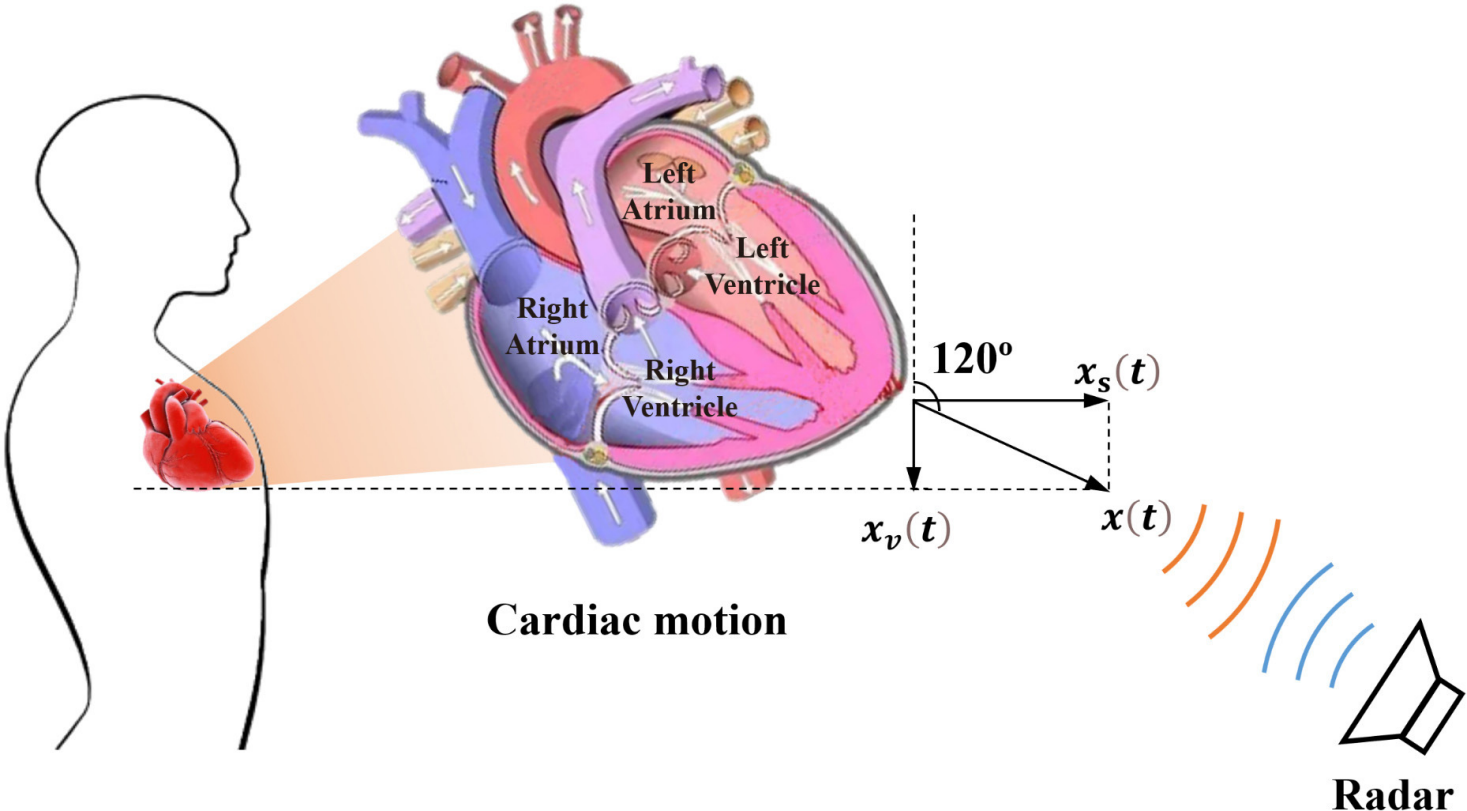
Schematic diagram of radar cardiac motion detection and the physiological structure of the heart.

The complex three-dimensional structure and its relative position in the thoracic cavity cause the heart to beat uniquely, making the observation results of heart pulsation vary greatly from different perspectives. As illustrated in Figure 1, the heart is located in the lower part of the anterior mediastinum of the thoracic cavity, and is wrapped with pericardium outside, about 2/3 on the left side of the anterior median line, and 1/3 on the right side. Heart contractions and relaxations cause the heart to twist from right to left along the long axis. In terms of mechanics, the longitudinal force of the myocardium impels the heart to vibrate (*x*_*s*_(*t*)) along the sagittal axis, and the transverse force contributes to shape changes (*x*_*v*_(*t*)) in the vertical axis direction. Consequently, the vector sum (*x*(*t*)) of the forces in three directions maximizes the amplitude of the heart movement in a certain direction in three-dimensional space. In addition, from an anatomical perspective, the heart consists of four parts: the left atrium, left ventricle, right atrium, and right ventricle, which cooperate to complete systemic circulation and pulmonary circulation. The key process is that the left ventricle pumps blood into the aorta and then transports it to all organs and tissues within the body; thus, the left ventricle beats most violently during this duration. However, the front heart is mostly blocked by the lung and pleura, and the other parts are also connected to adjacent organs and tissues, leaving only the apical part attached to the lower half of the sternum and left 4–6 costal cartilage. Consequently, the anatomical features described above resulted in movement of the apical part of the heart to be directly transmitted to the chest surface through the intercostal space, thus generating an obvious apical beat in the fifth intercostal space (7–9 cm to the left of the midline). In summary, based on the dynamic and static indicators, we speculate that there may be an optimal position and angle for cardiac pulsation observation in three-dimensional space.

**Figure 2:**
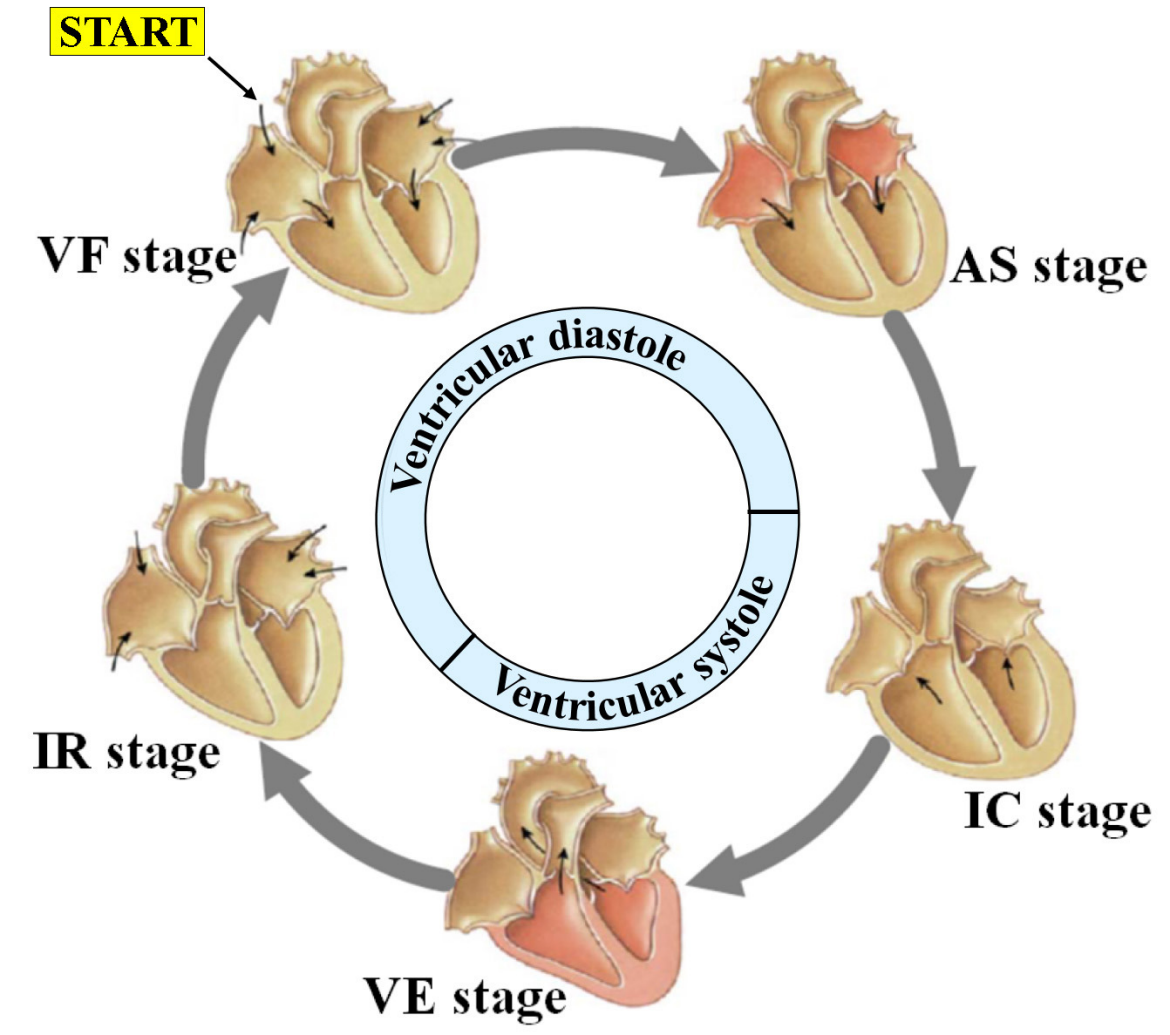
Motion characteristics during a cardiac cycle.

Driven by blood flow and electrical stimulation-induced myocardial contraction and relaxation, different chambers of the heart generate regular volume changes and micromotion rhythms at different stages during a single cardiac cycle, namely mechanical motion patterns. As shown in Figure 2, each cardiac motion cycle consists of five distinct stages including: (1) ventricular filling (VF), (2) atrial systole (AS), (3) isovolumetric ventricular contraction (IC), (4) ventricular ejection (VE), and (5) isovolumetric ventricular relaxation (IR). In the first stage, ventricular filling (VF) occurs when the semilunar valves (SV) are closed and the atrioventricular valves (AV) are open because the ventricular pressure is less than the atrial pressure. At this stage, the whole heart is relaxed, the blood in the atrium charges into the ventricle, and the ventricular filling accounts for 2/3 of the total filling, resulting in the rapid outward expansion of the heart. The second stage, atrial systole (AS), occurs when atria contract to pump their contained blood into ventricles, namely, the residual 1/3 ventricular filling. Although the heart contracts inward first because of the emptying of the atria, it expands outward immediately after because the extra blood in the atria is squeezed into the ventricles. The third stage, isovolumetric ventricular contraction (IC), occurs when the ventricles begin to contract and the SV/AV close. Although the ventricular pressure increases, no significant displacement occurs because there is no change in volume. Lastly, ventricular ejection (VE) occurs when the SV opens and ventricles contract and force blood into the arteries. This is because ventricular pressures rise to be higher than arterial pressures as the ventricle continues to contract, following which the SV is forced to open. During this process, the heart contracts inward because of the significant decrease in ventricular pressure and volume. During the fifth stage, isovolumetric ventricular relaxation (IR), ventricles finish the blood ejection, and SV/AV close when the ventricular pressure is lower than the aortic pressure and atrial pressure, respectively. The heart stops contracting and is relaxed, which ends the cycle. According to our analysis above, if we compare it with the electrophysiological activity of the heart, we speculate that there may be a certain correspondence between the mechanical activity and electrophysiological activity of the heart.

### 2.3 Analysis of the advantages of RCG and ECG cardiac physiological motion detection

**Figure 3:**
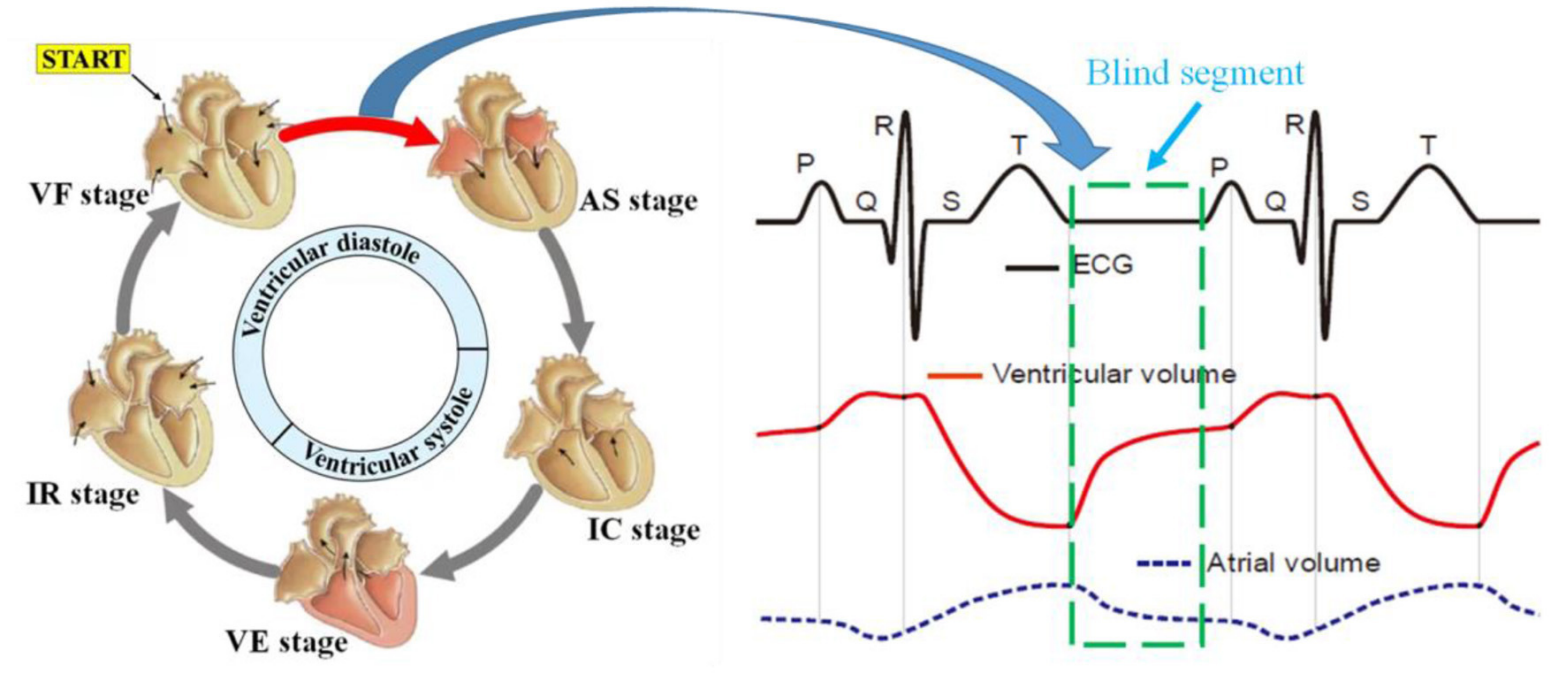
Rule of electrical activity and atrioventricular volume during a cardiac cycle.

The principle of ECG is to observe the change rule of cardiac current during a cardiac cycle, namely, the depolarization and repolarization processes of the atrial ventricle, which could help reverse the movement process and functional execution state of each chamber of the heart. Under the regulation of sympathetic and parasympathetic nerves, the heart transmits electrical signals generated usually by the sinoatrial node, atrioventricular node, and other nodes, to trigger heart muscle contraction, which in turn results in coordinated rhythmic contraction and relaxation of the heart throughout each cardiac cycle. Specifically, there is a special segment called the heart rest period between the rapid filling period and the atrial contraction period, similar to the interval marked by the green box in the ECG curve shown in Figure 3. Nevertheless, during heart blood flow, blood flow still occurs even during the heart rest period, so it still causes heart volume changes and mechanical motion, which undoubtedly contains (conveys) a large amount of information (abnormal heart disease) about the structure and function of the heart. Unfortunately, the ECG measurement method fails to detect the corresponding cardiac motion; thus, we refer to this interval as the blind segment.

As a novel non-contact measurement method, bio-radar mainly exploits the Doppler principle to measure the surface micro-motion caused by atrial ventricular contraction and relaxation movement transmitted to the body surface, namely the radar cardiogram. In other words, RCG measures cardiac mechanical motion instead of electrical activity. According to our analysis of the physiological characteristics of three-dimensional cardiac motion in Section Analysis of physiological characteristics of three-dimensional cardiac exercise, it can be guaranteed that the RCG could also contain similar information about the structure and function of the heart to the ECG and even detect cardiac mechanical motion during the *blind segment*. Therefore, the RCG holds two natural and critical superiorities to the ECG in the theory of non-contact and no-*blind segment*, which is expected to serve as a novel and refined measurement for whole-process cardiac detection.

### 2.4 Experimental scheme

**Figure 4:**
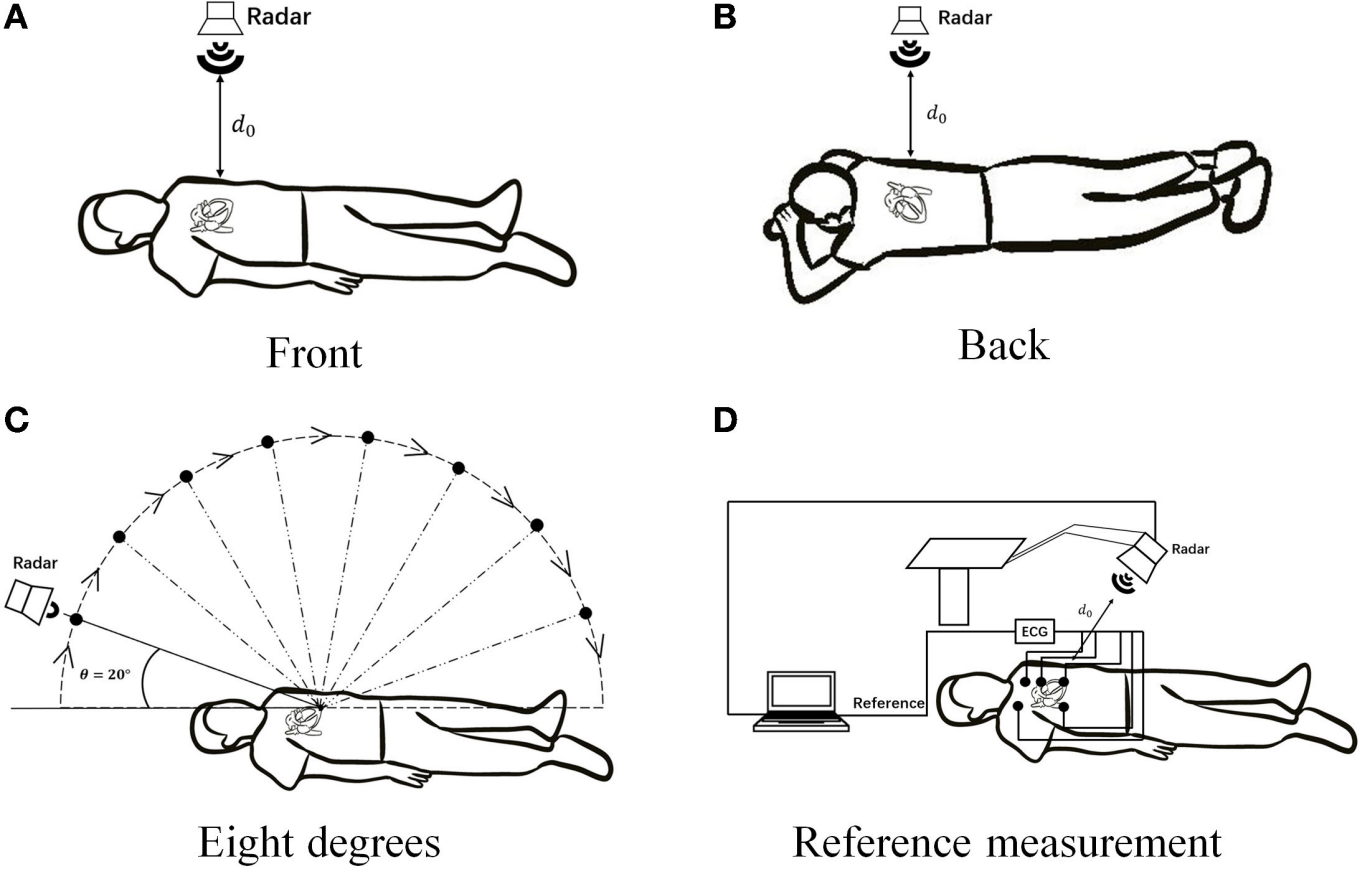
Schematic diagram of multiscale detection of a cardiac signal. **(A)** Front orientation measurement, **(B)** back orientation measurement, **(C)** along-the-arc measurements, and **(D)** reference measurement.

For multiscale measurements of cardiac motion, three types of experiments were designed along with coarse-to-fine scales, as shown in Figure 4. The experimental setup of anteroposterior measurements is illustrated in Figures 4A,B, and cardiac motion detection was performed from the front and back of the body. Second, cardiac signal detection from multiple perspectives of the front body experiment was performed, as shown in Figure 4C, which aimed to find the optimal position and angle for cardiac pulsation observation in a three-dimensional space. Finally, as illustrated in Figure 4D, contact (ECG) and non-contact (RCG) detections were used to simultaneously measure cardiac signals for performance comparison of the methods.

### 2.5 Cardiac signal acquisition system

**Figure 5:**
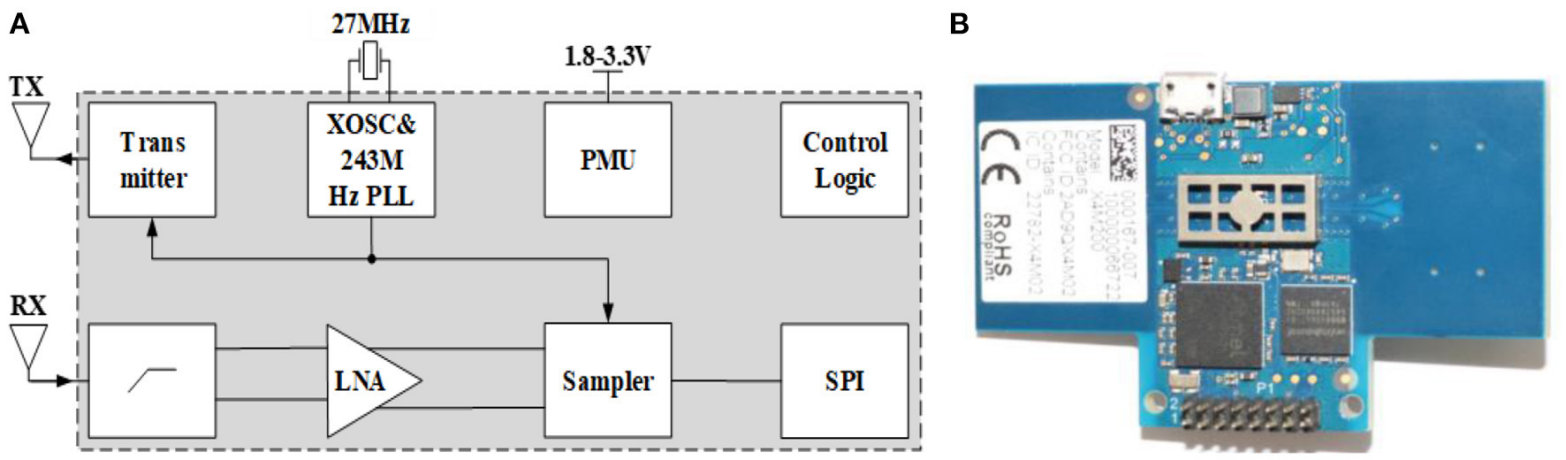
**(A)** Radar structure diagram and **(B)** radar object diagram.

The X4M200 pulse UWB radar system developed by Novelda was adopted in this study for human vital sign detection. The transmitting antenna of the radar adopts direct sampling technology and radio frequency (RF) interference suppression technology. The structure and radar system are shown in Figure 5, and its key parameters are listed in Table 1.

**Table 1:** Key parameters of the UWB radar system.

**Key parameters**	**Symbol**	**Value**
Center frequency	*f_*c*_*	7.29 GHz
Bandwidth	*B*	1.4 GHz
Pulse repetition rate	*PRR*	15.18 MHz
Detection range	*R*	0–9.9 m
Range point	*f_*r*_*	186
Scanning rate	*f_*s*_*	17 Hz

Similar to the system schematic in Figure 5, the transmitting antenna transmits pulses at a certain interval with a certain pulse repetition rate. After the pulse signal reaches the target, it is modulated and reflected by the target, and then received by the receiving antenna. Simultaneously, the system creates a frame of data that contains the motion information of the target. In a radar system, the phase-locked loop (PLL) of the transmitting antenna synthesizes the transmitting pulse. The front-end of the differential receiving antenna includes a high-pass filter (HPF), low-noise amplifier, and sampler for preliminary hardware filtering. A serial peripheral interface (SPI) was used to communicate with the host computer, and the power management unit (PMU) was responsible for the power supply of the radar system.

This radar system could operate under two modes with different bandwidths of 1.4 and 1.5 GHz respectively, and the corresponding center frequencies are 7.29 and 8.748 GHz, respectively. In our study, a mode with 7.29 a center frequency of 1.4 GHz bandwidth was chosen. It should be noted that the average output power (dBm/MHz) was >-44 dBm and the distance between human and radar antenna is about 0.55 m during measurement, thus the maximum power density of our bio-radar system is much lower than the accepted safe power density level of 10 mW/cm^2^, which would poses no threat to human health according to previous studies (22–24). Moreover, The Medical Ethic Committee of the First Affiliated Hospital of the Fourth Military Medical University approved the study. The informed consent of all subjects were obtained prior to volunteers' participation in the experiments.

## 3. Signal processing

**Figure 6:**
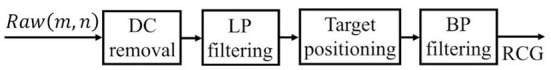
Processing flow chart of a cardiac radar signal.

Based on the fact that the reflected radar echo has been modulated by the chest movement, cardiac motion information can be obtained through a series of signal processing and demodulating on the radar echo. The signal processing flow chart of the cardiac radar signal shown in Figure 6 includes the following steps.

### 3.1 Preprocessing

The original time-range 2D radar echo signal contains DC components caused by static objects such as tables and ground, as well as the baseline drift of the echo caused by environmental factors, which cause strong interference in heartbeat extraction. In this study, the 100-order slide-window average subtraction method was used to remove the DC component and baseline drift, as shown in Equation (5):


(5) $$\begin{array}{l} R_{DC}\left(m,n\right)=Raw\left(m,n\right)-\frac{1}{100}\sum_{i=1}^{n+99}Raw\left(m,n\right) \end{array}$$

where *Raw*(*m, n*) is the raw data and *R*_*DC*_(*m, n*) is the radar echo after removing the DC and baseline drift.

Subsequently, a low-pass filter with a cutoff frequency of 5 Hz was used to filter out high-frequency noise interference to obtain mixed signals of respiration and heartbeat, as shown in Equation (6):


(6) $$\begin{array}{l} R_{LP}\left(m,n\right)=R_{DC}{\left(m,n\right)}^{*}H_{LP}\left(t\right) \end{array}$$

where *R*_*LP*_(*m, n*) is the data after removing the high-frequency noise and *H*_*LP*_(*t*) is the finite impulse response function of the low-pass filter.

**Figure 7:**
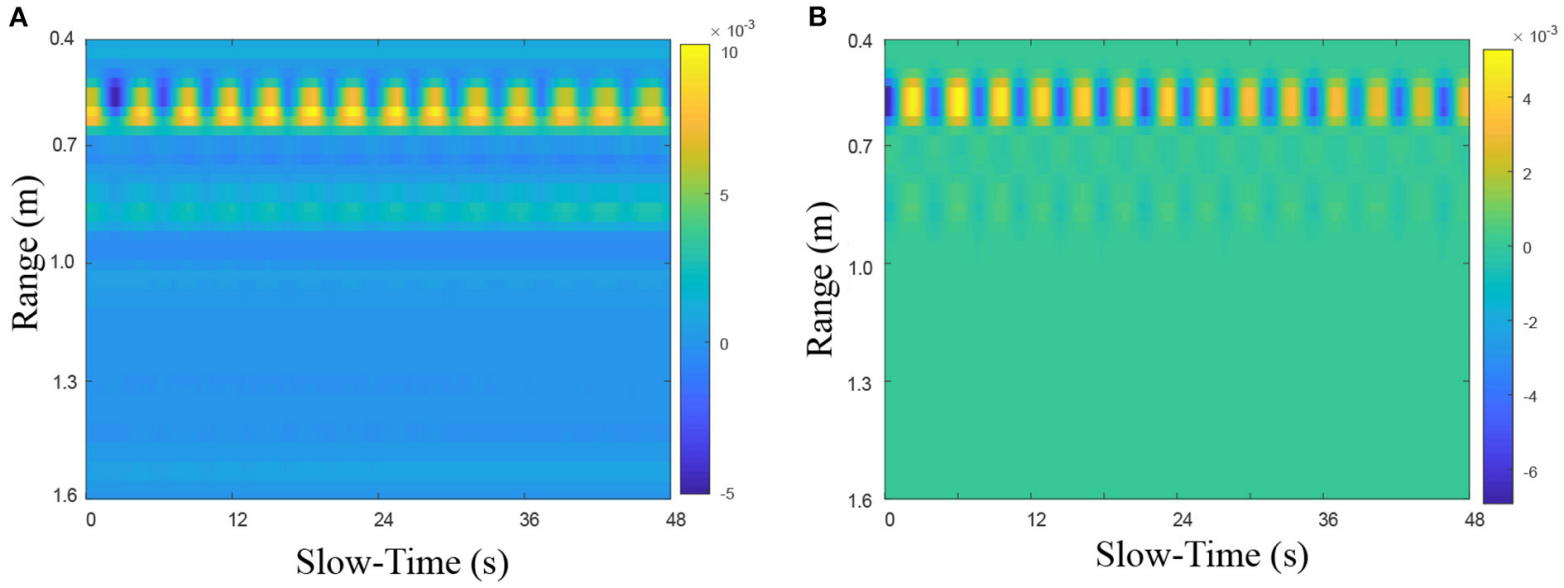
The preprocessing performance on heartbeat UWB radar echo **(A)** before and **(B)** after preprocessing.

Here, sample data were used to verify the effect of this preprocessing method. The time-range 2D radar echo (Figure 7A) was from a static lying human subject whose heart was directly in line with the UWB radar at a distance of 0.55 m. Obviously, except for the strong echo around the 0.55 m position, the 2D echo is also filled with various noise and interference. Nevertheless, this noise and interference can be removed effectively after preprocessing, as shown in Figure 7B.

### 3.2 Cardiac motion separation from radar cardiopulmonary physiological motion echo

For the time-range-preprocessed radar echo, the characteristics of IR-UWB allow cardiac motion information to exist in multiple range bins. Thus, before extracting cardiac signals, we must first select and locate the optimal range unit in which the human body lies. Here, the range bin with the maximum energy is selected as the optimal range bin signal, as shown in Equations (7) and (8):


(7) $$\begin{array}{l} S{\left(j\right)}_{j=1}=\sum_{n=1}^{n}{R^{2}}_{DC}\left(1,n\right) \end{array}$$

(8) $$\begin{array}{l} R_{TP}={\max\left[S\left(j\right)\right]}_{j=1}^{j=m} \end{array}$$

where ${R^{2}}_{DC}\left(1,n\right)$ is the slow time signal in the *j*th range bin, *S*(*j*)_*j* = 1_ is its energy sum, and *R*_*TP*_ is the optimal range bin signal.

Additionally, because the chest wall vibration detected by radar is a mixture of pulmonary motion (breathing) and cardiac motion (heartbeat), the next key step is to separate the heartbeat signal from the echo. The classic band-pass filter with the cut-off frequency of 0.85 and 3.3 Hz is exploited here, assuming the human heart rate to be 50–220 times per minute. The principle of this method can be expressed by Equation (9):


(9) $$\left\{ \begin{array}{l} R_{BP}=R_{TP^{*}}H_{BP}(t)\\ R_{LP}=R_{TP^{*}}H_{LP}(t) \end{array}\right.$$

where *R*_*BP*_is the obtained cardiac signal, *H*_*BP*_(*t*) is the finite impulse response function of the bandpass filter.*R*_*LP*_ is the obtained respiration signal, and *H*_*LP*_(*t*) is the finite impulse response function of the low-pass filter.

## 4. Experiments and results

### 4.1 Experimental setup

According to the anatomical structure of the heart, cardiac apex motion, such as systole, diastole, or torsion, is conducted through the gap between the fourth and fifth ribs to generate micro-movement at the skin surface. Therefore, it is reasonable to speculate that there is an optimal observation position for micromotion signals. Clinically, the fifth intercostal space can be localized using one notch counting down from the fourth intercostal space, which is located on the line connecting the two nipples. The skin surface area of the micro-movement originating from the heartbeat is an approximate circle with a diameter of 2–2.5 cm. This circular area is the right position that needs to be aimed at by the radar.

Then, anteroposterior and arc measurements were carried out. Radar cardiac signals of five male subjects aged 24–43 years were collected. For anteroposterior measurements, human subjects lay on the ground in a supine or prone position while holding their breath. The radar was placed 55 cm away from the human body. Eight traits of 10-s signal for each person were recorded, four collected from the front side and four from the back side.

The along-the-arc measurement was designed to determine the optimal observation angle that could ensure the acquisition of the best radar echo signal. To avoid inconsistencies caused by side-lobe energy attenuation or further distance energy attenuation, an equal-radius measuring method is proposed to maintain the detection distance between the body surface area of the cardiac apex and the radar constant. A laser pointer was used to ensure that the radar is always aimed at the cardiac apex. Each human target was detected eight times along an arc in the sagittal plane with the circle center of the cardiac apex and an angle step of 20°each time. The along-the-arc measurement ensured that the best radar echo signal was obtained at each observation angle.

Due to support from the ground, body shaking was minimal when the human target is measured in a lying posture. In this study, the lying position was used for cardiac signal collection. Respiratory harmonics cause serious interference to heartbeat signals (respiratory harmonics could be close or even coincide with the cardiac signal in the frequency domain), which were difficult to remove. To eliminate this harmonic interference, the cardiac signals were recorded under breath-holding conditions.

### 4.2 Anteroposterior measurements

**Figure 8:**
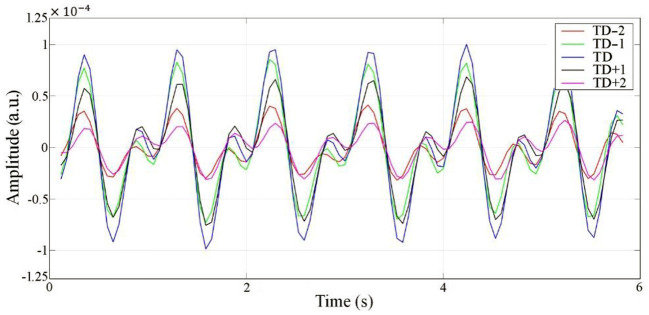
RCG results from five adjacent range bins.

Radar echo data are a 2-dimensional array alone with range (fast time) and slow time, which can be divided into a limited number of range bins along the range dimension. If the motion amplitude of the target is beyond the width of a single range bin, the motion will appear in several adjoining range bins and influence the neighbors, which is called the range bin effect (25). Therefore, an experiment was performed to explore whether the proposed radar has a range-bin effect. Five range bins, TD−2, TD−1, TD, TD+1, and TD+2, centered symmetrically on the range bin signal with maximum energy were collected. According to the results, no morphological differences were observed, except for the difference in amplitude among the five collected range bins. This demonstrates that there is no range-bin effect, and the cardiac motion signal at the range point with the largest energy can represent the overall range bins. The cardiac signals of the five range bins are shown in Figure 8.

**Figure 9:**
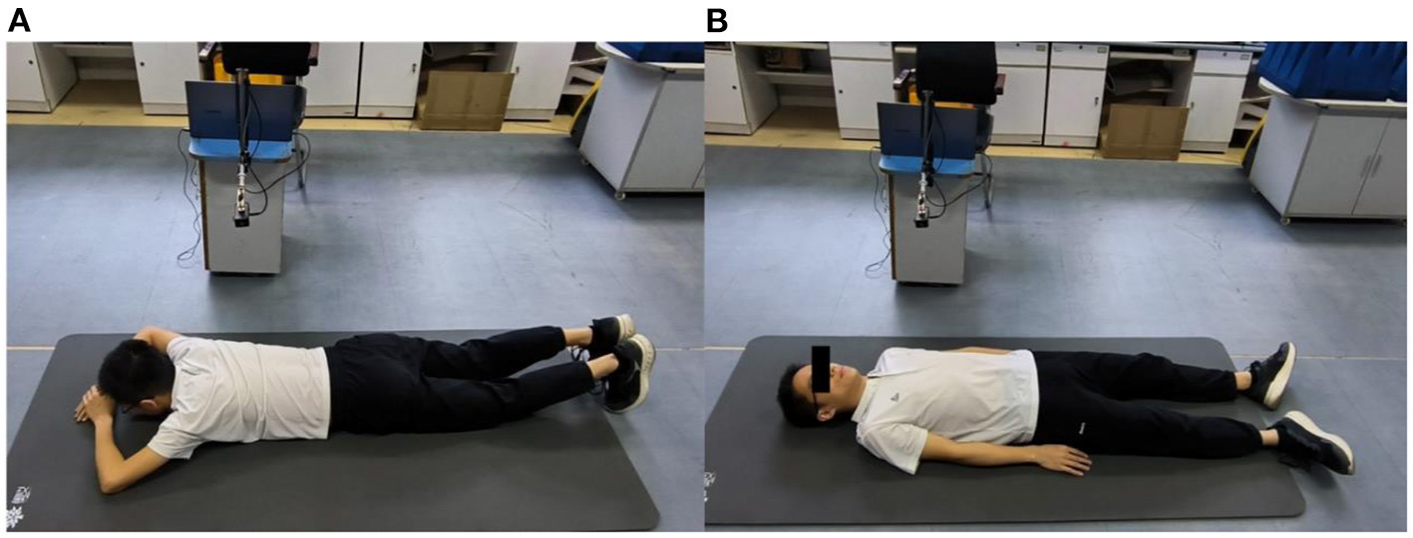
Anteroposterior measurements, **(A)** measured from back side, **(B)** measured from front side.

To study the detection performance from the front and back orientations, we collected the radar cardiac signals of five male subjects who held their breath while lying in a supine or prone position. As shown in Figure 9, 40 groups of eight traits for each person were collected under the anteroposterior measurement scenario. Li et al. (20) found that the energy ratio of heartbeat to respiration detected from the back was larger than that from the front, cardiac signal feature extraction for disease diagnosis requires critical characteristics of large amplitude and more detailed information.

**Figure 10:**
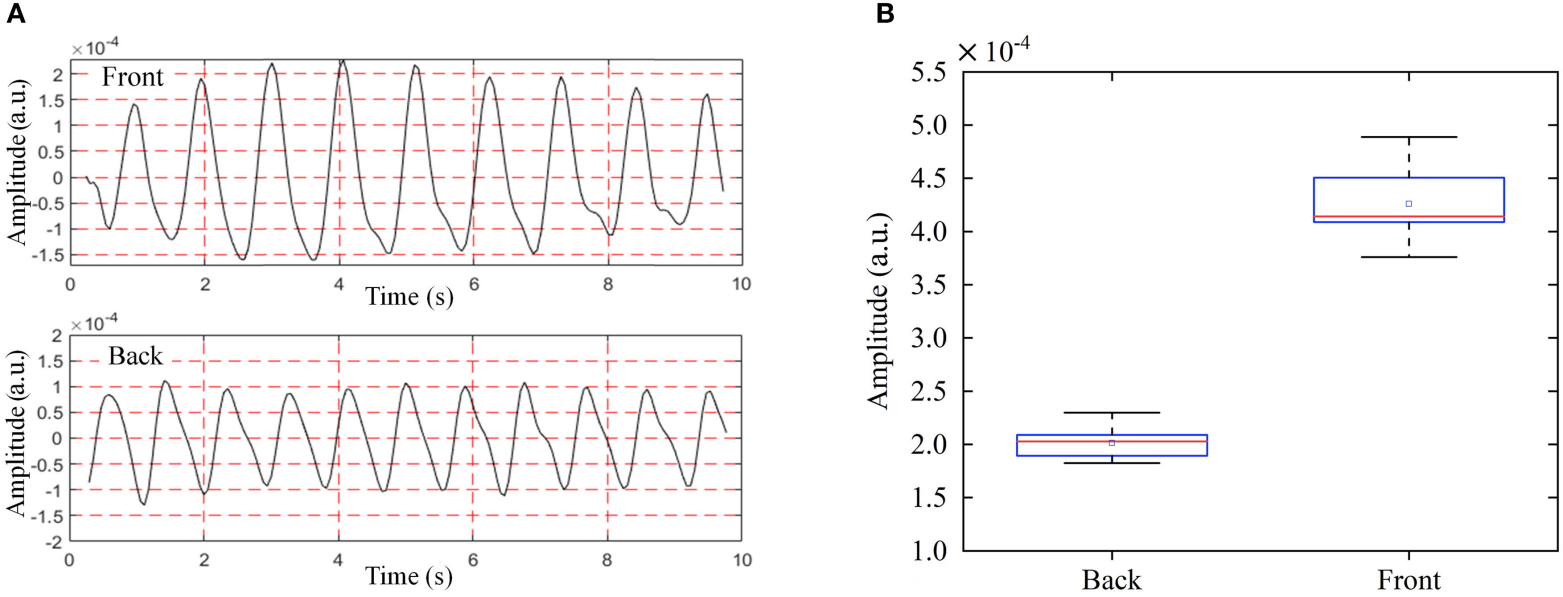
RCG results of anteroposterior measurements from the back and front side, **(A)** time domain RCG amplitude results, **(B)** box plot of RCG amplitude results.

The measurement results from the front side and back side are shown in Figure 10. The median of all RCG time-domain amplitudes detected from the front side was 3.67 × 10^−4^ and the standard deviation (STD) was 4.05 × 10^−6^. The median and STD of the RCG amplitude from the back side were 1.53 × 10^−4^ and 4.04 × 10^−6^ respectively. We can see that the RCG amplitude detected from the front side was larger than that from the back side, and superiority also existed with respect to the detailed information. The reasons for this are as follows: The cardiac movement just needs to pass through the fifth intercostal space to reach the skin surface and be detected by radar from the frontal detection perspective when the attenuation is weak. However, from the backside detection view, cardiac motion needs to be conducted through the spine, lungs, muscles, skin, and other tissues and organs, and then detected by radar, so the attenuation was much greater than that of front side detection.

### 4.3 Along-the-arc measurements

**Figure 11:**
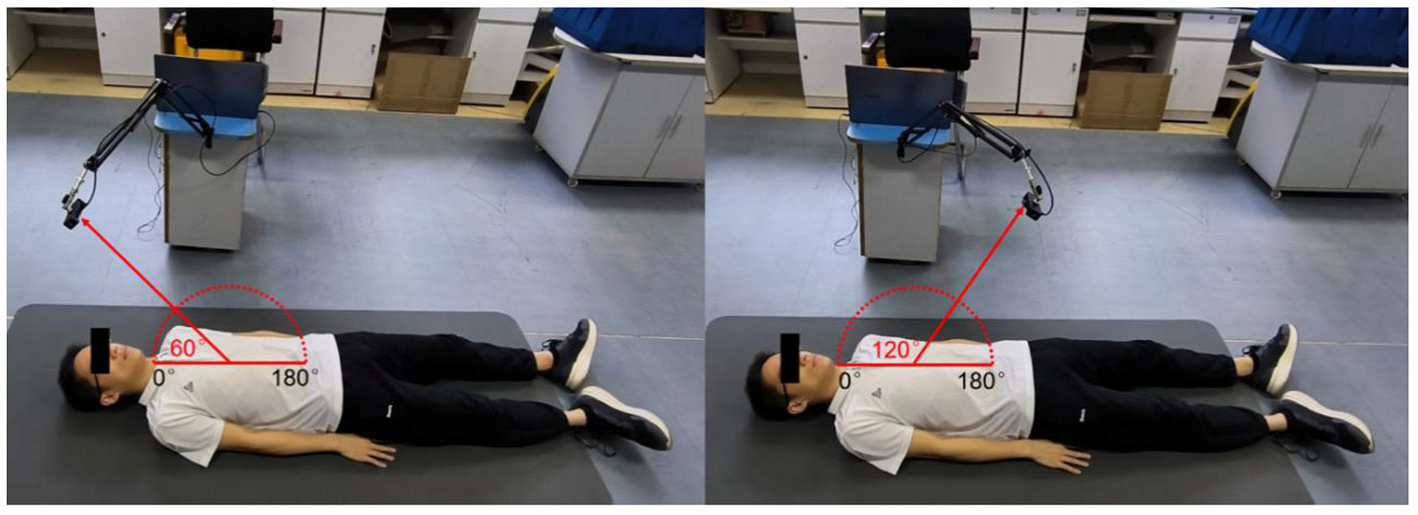
Scenarios of the along-the-arc measurements.

**Figure 12:**
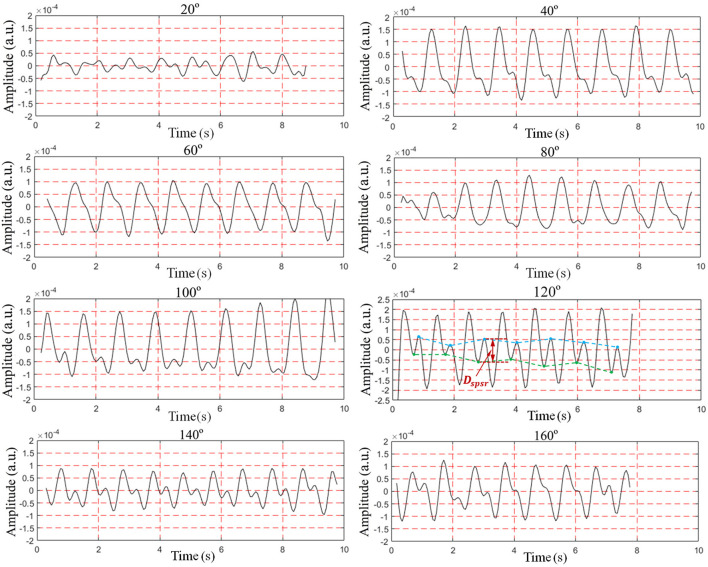
RCG results at different angles.

**Figure 13:**
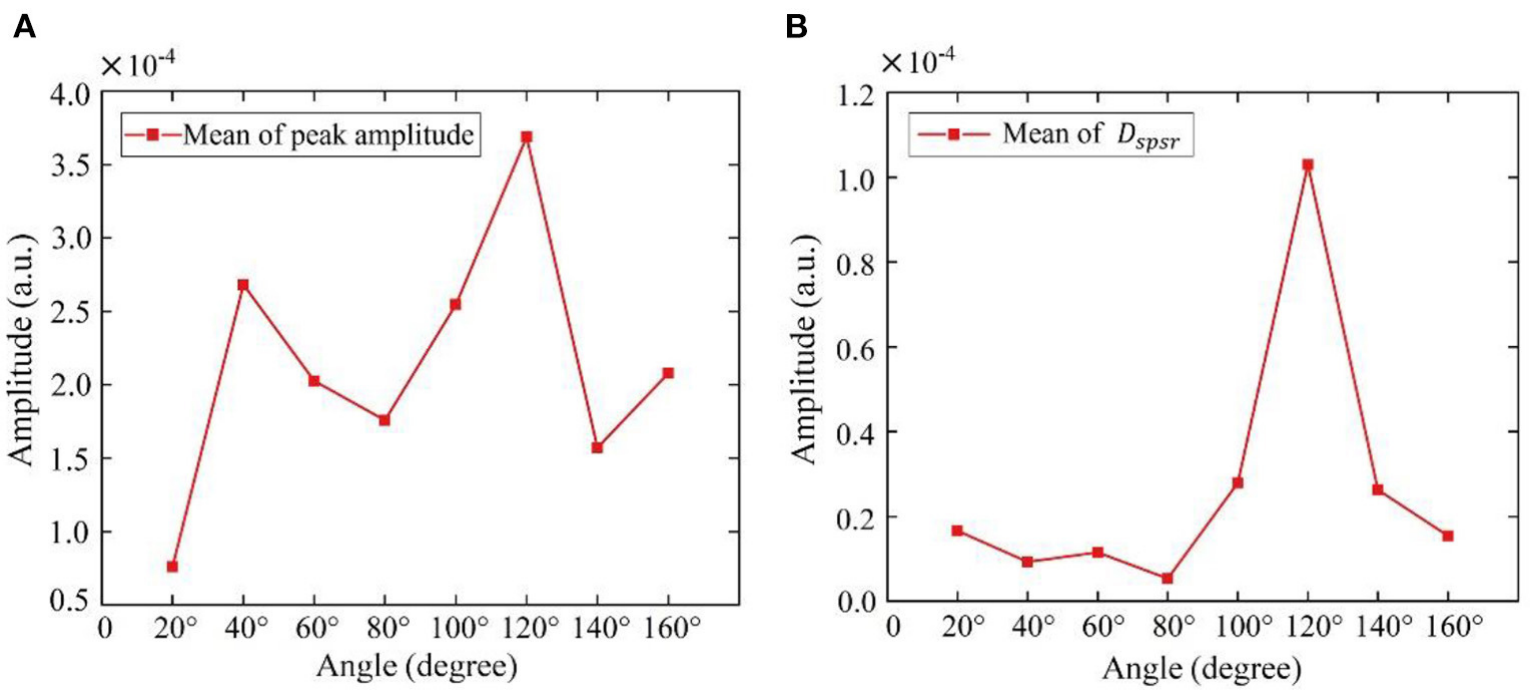
Results of RCG at different angles from front side **(A)** overall results (mean of peak amplitude), **(B)** detailed results (mean of *D*_*spsr*_).

To find the optimal detection angle of the view from the front side, measurements from eight different angles were carried out when the human target was lying in a supine posture and holding their breath. The angles, 20, 40, 60, 80, 100, 120, 140, 160°, within sagittal plane, were adopted. The radar changed the angle along an arc with a radius of 55 cm. The scenario is shown in Figure 11. Each person at each angle was detected once, and five subjects amounted to 40 sets of data. The results are shown in Figures 12, 13. The best cardiac signal was observed at 120° both in amplitude and morphology. When the radar detection angle increasing from 20 to 160°, the amplitudes, representing echo energy, gradually increased and then decreased. The RCG reached its maximum energy at an angle of 120°. Morphologically, the average difference between the second peak and second trough in RCG cardiac cycles, denoted as *D*_*spsr*_, moved from unobvious to obvious and then unobvious again along with the increase in the detecting angle. As shown in Figure 13, the second most obvious peak also appeared at 120°. The RCG results, both in energy and morphology, indicated that the vector sum of the three-dimensional motion of the heart was in the direction of 120°.

### 4.4 Comparison of diastasis measurements between RCG and ECG

**Figure 14:**
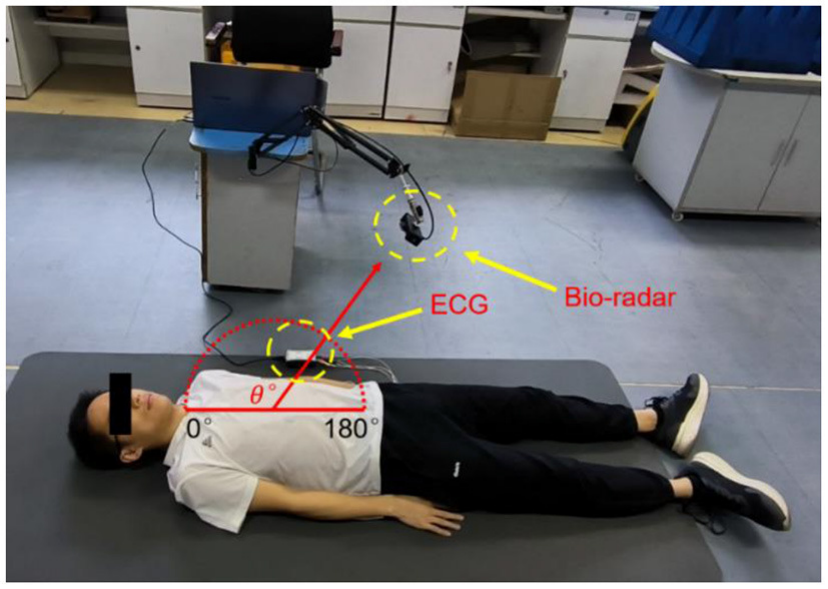
Synchronous acquisition experiment of RCG and ECG.

**Figure 15:**
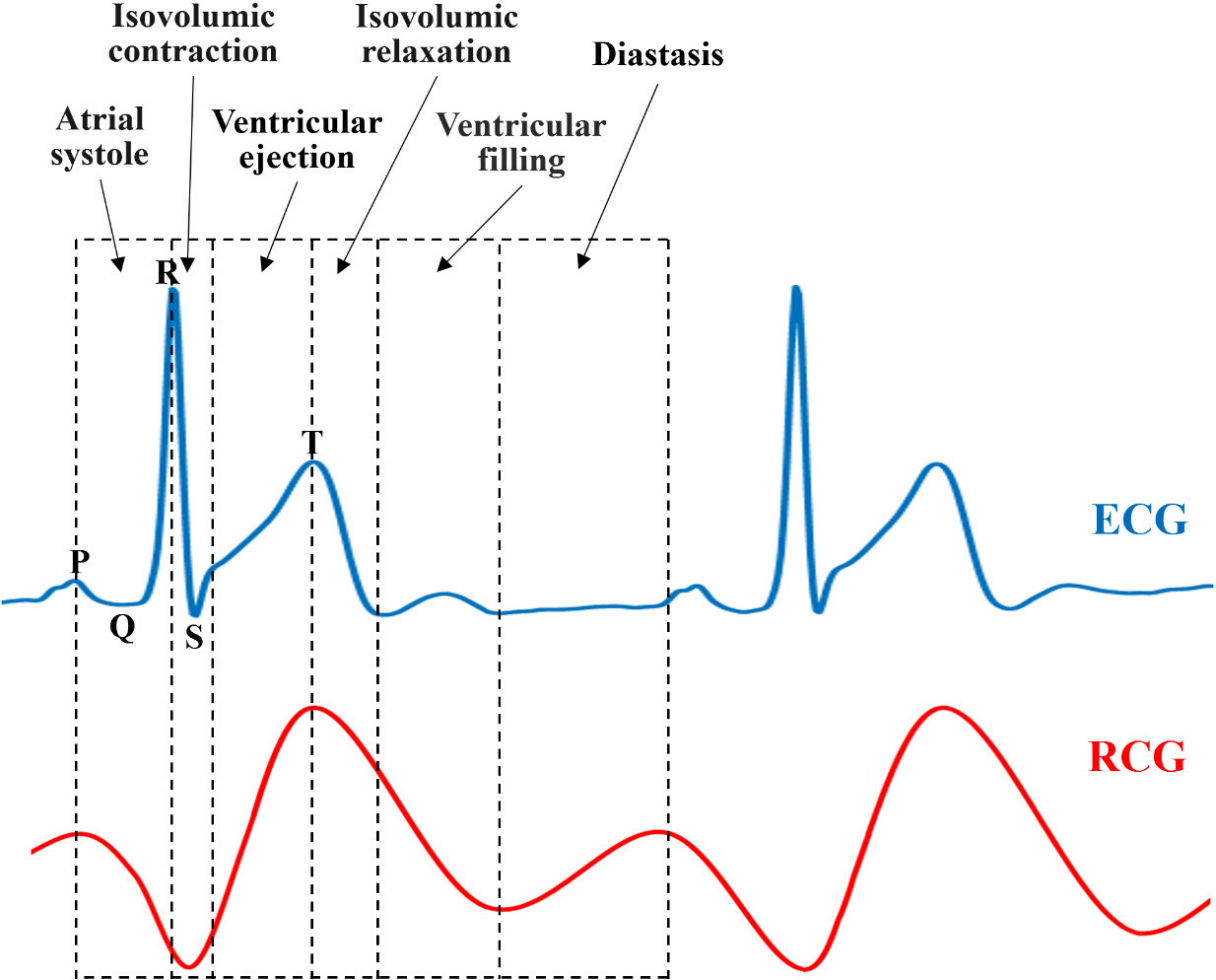
Comparison of RCG and ECG.

To study the similarities and differences between the RCG and ECG, simultaneous acquisition experiments using a radar sensor and ECG are illustrated in Figure 14. A comparative analysis of the features and cardiac cycle staging between ECG and RCG is shown in Figure 15. The maximum RCG value indicated that ventricular relaxation corresponded to the T wave in the ECG. The stage after this maximum value was the change in ventricular volume from small to large. The second peak of the RCG represents the start of atrial contraction and corresponds to the P wave in the ECG. The stage related to the time interval from second peak to second trough was the change in atrial volume from large to small.

With regard to the ECG results in Figure 15, there is a period after the stage of ventricular filling before the next P-wave presents a straight line without any fluctuation, which is called diastasis. During diastasis, the heart maintains systole and diastole phases, and the heart volume keeps changing, which means that the heart still has motion in the diastasis period. The motion in diastasis is transmitted to the body surface and detected by the radar, which appears as a fluctuation in the RCG. Therefore, the RCG can detect and obtain cardiac motion information from a blind segment that has no ECG information. Considering that the cardiac motion signal in the blind area contains information on heart motion status and disease, RCG contains more information than ECG and can provide real-time whole-cardiac cycle health monitoring.

The position of the cardiac apex beat is affected by various physiological and pathological factors. For example, when the human body is in a lying position, the surface beating position where the cardiac motion is conducted changes with different lying postures. Furthermore, different body shapes, enlargement of the heart, and displacement of the mediastinum and diaphragm also affect the surface-beating position. Research on disease diagnosis based on radar cardiac motion signals is still in its infancy. In practical application scenarios, the human breathing signal causes severe interference with the RCG. Body shaking is another great challenge to RCG measurement that needs to be suppressed. In addition, the correspondence between RCG features and cardiac pathological changes needs to be studied further. Further research should be conducted to address these issues.

## 5. Conclusion

This study systematically investigated the influence of the detecting position, orientation, and angle on cardiac motion measurements. Considering the anatomical position, posture, physiological structure, and motion characteristics of the atrium and ventricle, multiscale measurements were designed and performed. The best location, optimal orientation, and angle of detection were first found and experimentally verified. In anteroposterior measurements, a better RCG amplitude was obtained when the radar was aimed at the fifth intercostal space and illuminated the human body from the front side. For along-the-arc measurements, an optimal RCG result was observed with a detection angle of 120° both in overall amplitude and detailed information, which means that the vector sum of three-dimensional cardiac motion is in the direction of 120°. It is worth mentioning that, some non-absolute-amplitude-based-features of RCG, such as time interval between two points, relative amplitude and magnitude ratio, etc. are also important diagnostic basis for heart disease and these features do not require high accuracy detection angle. Furthermore, due to differences in detection theory, RCG can detect information in the blind area (diastasis) where ECG cannot.

The new discoveries of this study lay a theoretical foundation for RCG measurements and are of great significance for RCG applications in cardiovascular disease diagnosis. This could also serve as a foundation for subsequent RCG-based studies.

## Data availability statement

The raw data supporting the conclusions of this article will be made available by the authors, without undue reservation.

## Ethics statement

The studies involving human participants were reviewed and approved by the Medical Ethics Committee of the First Affiliated Hospital of the Fourth Military Medical University. The patients/participants provided their written informed consent to participate in this study. Written informed consent was obtained from the individual(s) for the publication of any potentially identifiable images or data included in this article.

## Author contributions

Conceptualization and funding acquisition: YZ and J-qW. Methodology, writing, and original draft preparation: J-hQ, F-gQ, and YZ. Investigation and data: F-gQ, JM, and F-lL. Writing, review, and editing: Y-hQ, XY, and QA. Visualization: DS, HL, and H-jX. Supervision: K-dY and YZ. All authors have read and agreed to the published version of the manuscript.
